# Supplementary material for: Segmental Evaluation of Thoracic Aortic Calcium and Their Relations with Cardiovascular Risk Factors in the Brazilian Longitudinal Study of Adult Health (ELSA-Brasil)
Source: Cells. 2021 May 18;10(5):1243. doi: 10.3390/cells10051243 (PMC8158124; doi:10.3390/cells10051243)
Supplement: Supplementary file 1 [file cells-10-01243-s001.zip › cells-1179727-SI.pdf]

cells

MDPI

Supplementary Materials

Table S1. Blood pressure and other cardiovascular risk factors associated with the presence of calcium in each aortic thoracic segments in the final multivariate analysis. ELSA-Brasil, 2015–2016.

Variables	AAC > 0	ATAC > 0	DTAC > 0	ADTAC > 0
Age (*years*)	**1.11 (1.10–1.13) ‡**	**1.09 (1.07–1.11) ‡**	**1.11 (1.09–1.12) ‡**	**1.11 (1.09–1.12) ‡**
Women	0.95 (0.78–1.16)	1.16 (0.93–1.44)	0.87 (0.71–1.06)	0.97 (0.80–1.18)
Educational level				
University degree	1.00	1.00	1.00	1.00
Complete secondary	1.15 (0.92–1.44)	1.00 (0.78–1.28)	0.99 (0.79–1.25)	1.04 (0.83–1.30)
Complete elementary	1.72 (0.98–3.02)	0.91 (0.56–1.49)	0.90 (0.57–1.43)	0.79 (0.50–1.25)
Incomplete elementary	1.66 (0.91–3.04)	**1.68 (1.02–2.78) ***	1.60 (0.97–2.64)	**1.99 (1.17–3.36) ***
Smoker				
Never	1.00	1.00	1.00	1.00
Past	**1.26 (1.01–1.58) ***	**1.80 (1.42–2.27) ‡**	0.94 (0.75–1.18)	1.16 (0.93–1.43)
Current	**2.09 (1.47–2.95) ‡**	**2.55 (1.81–3.60) ‡**	**1.79 (1.30–2.48) ‡**	**1.90 (1.38–2.61) ‡**
Physical activity				
Insufficient	1.00	1.00	1.00	1.00
Moderate	1.21 (0.94–1.54)	**1.36 (1.05–1.76) ***	0.85 (0.66–1.09)	0.96 (0.75–1.22)
Vigorous	1.01 (0.72–1.42)	1.30 (0.87–1.94)	1.23 (0.85–1.78)	1.27 (0.89–1.81)
Family history of CVD	**1.37 (1.12–1.68) †**	1.17 (0.94–1.45)	1.07 (0.87–1.31)	1.13 (0.93–1.39)
Body mass index (*kg/m*²)	**1.05 (1.03–1.07) ‡**	**1.06 (1.03–1.08) ‡**	**1.04 (1.02–1.06) ‡**	**1.0 (1.0–1.1) ‡**
Systolic blood pressure (*mmHg*)	1.01 (1.00–1.02)	1.01 (1.00–1.02)	**1.02 (1.01–1.03) ‡**	**1.01 (1.00–1.03) ***
Diastolic blood pressure (*mmHg*)	1.00 (1.00–1.01)	1.00 (0.98–1.02)	1.00 (0.98–1.01) ‡	1.00 (0.99–1.02)
Dyslipidemia	1.17 (0.96–1.43)	**1.70 (1.37–2.10) ‡**	**1.26 (1.03–1.53) ***	**1.48 (1.22–1.79) ‡**
Use of blood pressure lowering medications	**1.56 (1.25–1.93) ‡**	**1.52 (1.22–1.91) ‡**	**1.82 (1.48–2.25) ‡**	**1.82 (1.49–2.23) ‡**
Diabetes	1.06 (0.79–1.41)	**1.31 (1.00–1.71) ***	1.28 (0.99–1.66)	**1.42 (1.09–1.84) †**

Data are expressed as odds ratio (95% confidence interval). * $p \leq 0.05$; † $p \leq 0.01$; ‡ $p \leq 0.001$. AAC indicates aortic arch calcium; ATAC, ascending thoracic aortic calcium; CVD, cardiovascular disease; DTAC, descending thoracic aortic calcium; ADTAC, ascending plus descending thoracic aortic calcium.

Table S2. Cardiovascular risk factors associated with the presence of calcium in the thoracic aortic segments after adjustments for body surface in the final multivariate analysis. ELSA-Brasil, 2015–2016.

Variables	AAC > 0	ATAC > 0	DTAC > 0	ADTAC > 0
Age (years)	**1.12 (1.10–1.13) ‡**	**1.09 (1.08–1.11) ‡**	**1.11 (1.10–1.13) ‡**	**1.11 (1.10–1.13) ‡**
Women	0.86 (0.65–1.14)	1.11 (0.82–1.52)	0.76 (0.57–1.01)	0.87 (0.66–1.15)
Educational level				
University degree	1.00	1.00	1.00	1.00
Complete secondary	1.15 (0.92–1.44)	1.01 (0.78–1.30)	1.01 (0.80–1.27)	1.06 (0.85–1.32)
Complete elementary	1.73 (0.98–3.04)	0.93 (0.57–1.51)	0.92 (0.58–1.46)	0.81 (0.51–1.28)
Incomplete elementary	1.62 (0.89–2.97)	**1.79 (1.08–2.94) ***	**1.68 (1.02–2.77) ***	**2.04 (1.21–3.44) †**
Smoker				
Never	1.00	1.00	1.00	1.00
Past	**1.26 (1.01–1.57) ***	**1.79 (1.42–2.26) ‡**	0.93 (0.74–1.16)	1.15 (0.93–1.42)
Current	**2.08 (1.47–2.94) ‡**	**2.53 (1.80–3.56) ‡**	**1.77 (1.28–2.44) ‡**	**1.87 (1.36–2.57) ‡**
Physical activity				
Insufficient	1.00	1.00	1.00	1.00

Moderate	1.22 (0.95–1.55)	**1.36 (1.05–1.76) ***	0.85 (0.67–1.10)	0.95 (0.75–1.21)
Vigorous	1.03 (0.74–1.45)	1.32 (0.89–1.98)	1.27 (0.88–1.83)	1.29 (0.91–1.84)
Family history of CVD	**1.37 (1.12–1.68) †**	1.18 (0.95–1.47)	1.09 (0.88–1.33)	1.15 (0.94–1.40)
Body mass index (kg/m2)	**1.06 (1.03–1.10) ‡**	**1.06 (1.03–1.10) ‡**	**1.05 (1.02–1.09) ‡**	**1.06 (1.02–1.09) ‡**
Body surface (m^2)	0.88 (0.69–1.13)	0.98 (0.75–1.28)	0.89 (0.69–1.14)	0.92 (0.72–1.17)
Dyslipidemia	1.20 (0.98–1.46)	**1.73 (1.40–2.15) ‡**	**1.29 (1.06–1.58) ***	**1.51 (1.25–1.84) ‡**
Hypertension	**1.52 (1.24–1.88) ‡**	**1.62 (1.30–2.03) ‡**	**2.09 (1.71–2.57) ‡**	**1.96 (1.61–2.39) ‡**
Diabetes	1.06 (0.80–1.42)	**1.34 (1.03–1.75) ***	**1.33 (1.03–1.71) ***	**1.45 (1.12–1.88) †**

Data are expressed as odds ratio (95% confidence interval). * $p \le 0.05$; † $p \le 0.01$; ‡ $p \le 0.001$. AAC indicates aortic arch calcium; ATAC, ascending thoracic aortic calcium; CVD, cardiovascular disease; DTAC, descending thoracic aortic calcium; ADTAC, ascending plus descending thoracic aortic calcium.
